# Clinical Phenotypes of *CDHR1*-Associated Retinal Dystrophies

**DOI:** 10.3390/genes13050925

**Published:** 2022-05-22

**Authors:** Volha V. Malechka, Catherine A. Cukras, Emily Y. Chew, Yuri V. Sergeev, Delphine Blain, Brett G. Jeffrey, Ehsan Ullah, Robert B. Hufnagel, Brian P. Brooks, Laryssa A. Huryn, Wadih M. Zein

**Affiliations:** Ophthalmic Genetics and Visual Function Branch, National Eye Institute, National Institutes of Health, Bethesda, MD 20892, USA; omalechko@gmail.com (V.V.M.); cukrasc@nei.nih.gov (C.A.C.); echew@nei.nih.gov (E.Y.C.); sergeevy@nei.nih.gov (Y.V.S.); dblain@mail.nih.gov (D.B.); brett.jeffrey@nih.gov (B.G.J.); ehsan.ullah@nih.gov (E.U.); robert.hufnagel@nih.gov (R.B.H.); brooksb@nei.nih.gov (B.P.B.); laryssa.huryn@nih.gov (L.A.H.)

**Keywords:** *CDHR1*, retinal dystrophy, autosomal recessive retinal dystrophies

## Abstract

The retinal dystrophy phenotype associated with *CDHR1* retinopathy is clinically heterogenous. In this study, we describe the clinical and molecular findings of a retinal dystrophy cohort (10 patients) attributed to autosomal recessive *CDHR1* and report novel variants in populations not previously identified with *CDHR1*-related retinopathy. Seven patients had evaluations covering at least a three-year period. The mean age of individuals at first symptoms was 36 ± 8.5 years (range 5–45 years). Visual acuity at the last visit ranged from 20/20 to 20/2000 (mean LogMAR 0.8 or 20/125). Three clinical subgroups were identified: rod–cone dystrophy (RCD), cone–rod dystrophy (CRD), and maculopathy. Extinguished scotopic electroretinography responses were noted in the RCD patients. Macular involvement was noted in all patients and documented on color fundus photography, fundus autofluorescence, and optical coherence tomography. Notable asymmetry of the degree of macular atrophy was present in two patients. The possible association between *CDHR1* variants and clinical findings was predicted using molecular modeling.

## 1. Introduction

Autosomal recessive retinal dystrophies are genetically heterogeneous and often associated with loss of function of cone and rod photoreceptors, potentially leading to blindness [1,2,3]. This group of conditions is associated with variants in around 150 genes encoding proteins responsible for the functional and structural integrity of the retina (RetNet: summary of genes and loci causing recessive retinal dystrophies). The cadherin-related family member 1, also known as protocadherin 21, or photoreceptor-specific cadherin, is a structural transmembrane photoreceptor protein that localizes at the base of cone and rod photoreceptor outer segments and plays an essential role in the maintenance of the structure and survival of photoreceptors [4]. It is encoded by *CDHR1*, a gene located on chromosome 10q23.1 [5]. 

The genomic structure of *CDHR1* was first reported in 2005 [6], and mutations in this gene cause retinal dystrophies [7]. In *prCAD^−^*^/−^ mice with dysfunctional photoreceptor-specific cadherin, the outer segments of photoreceptors are disorganized and shorter in size, and progressive apoptotic death of photoreceptor cells is noted from one month of age [4]. *Cdhr1^−/−^* mice exhibit outer retinal thinning on optical coherence tomography (OCT) at 1 month of age with progressive degeneration through 15 months. Dark-adapted and light-adapted flicker electroretinography (ERG) identify severe functional deficits in *Cdhr1^−/−^* mice at 1 month of age [8]. These early functional deficits affecting rod and cone photoreceptors with relatively mild changes in the retinal structure reflect the human phenotype of *CDHR1*-associated retinal degeneration. In humans, variants in *CDHR1* are associated with autosomal recessive cone–rod dystrophies (CRD) [9,10]; rod–cone dystrophies (RCD) [11]; and as recently described, late-onset macular degeneration (LOMD) [12,13]. Patients with *CDHR1* variants demonstrate variability in disease onset (range 5–45 years) and severity.

Missense, nonsense, and copy-number variants in the *CDHR1* gene have been reported to cause retinal dystrophy. Missense mutations can lead to perturbations of a protein structure and impact normal protein function. The effect of a missense mutation on structural changes can be analyzed in silico by molecular modeling. Here, we introduce a molecular modeling approach to suggest the possible association between predicted alteration or damage to the structure of CDHR1 protein, and severity of retinal phenotype.

## 2. Materials and Methods

### 2.1. Patient Identification

Patients who were examined at the National Eye Institute (NEI, Bethesda, MD, USA) between December 2006 and January 2021 and had molecular results indicating *CDHR1*-associated dystrophy were included in this study.

### 2.2. Molecular Diagnosis

Clinical genetic testing was performed by Next Generation Sequencing (NGS) panels of retinal dystrophy genes. All detected variants were classified according to the guidelines of the American College of Medical Genetics and Genomics (ACMG) [14]. Segregation analysis was offered but was not performed since first-degree relatives were unavailable for testing.

### 2.3. Clinical Assessment

All patients underwent detailed clinical evaluations by inherited retinal disease specialists at the NEI. A complete medical history was obtained including pertinent ophthalmic information and known family history. Electronic medical records were the sources of data collection and analysis. Visual function testing, ophthalmic imaging, and ERG findings were reviewed. The evaluation of best corrected visual acuity (BCVA) was performed using Snellen acuity charts. Visual acuity assessments were standardized and converted to the Logarithm of the Minimum Angle of Resolution (LogMAR) unit values using the formula LogMAR = −Log (Decimal Acuity) [15]. The assessment of visual fields was performed using Goldmann kinetic perimetry with V4e, I4e, and I1e isopters. Color discrimination was measured monocularly with the Farnsworth D-15 dichotomous test. Full-field scotopic and photopic ERG responses were obtained after 30 min of dark adaptation and 10 min of light adaptation, respectively, using contact lens electrodes (Burian-Allen; Hansen Ophthalmic Development Laboratory Inc, Bellingham, WA, USA). ERG procedures were performed according to the International Society for Clinical Electrophysiology of Vision (ISCEV) standards [16]. The presence of atrophic and pigmentary changes, and the health of retinal vasculature and the optic nerve was documented through color photography and the fundus autofluorescence (FAF) imaging system (Topcon Corporation, Tokyo, Japan). The OCT imaging system (Spectralis Heidelberg Engineering LTD; Cirrus HD OCT, Carl Zeiss Meditec, Germany) was used to evaluate total retinal thickness, outer nuclear layer (ONL) thickness, as well as cross-sectional structural changes.

### 2.4. Molecular Modeling

The structure of the human CDHR1 protein was modeled using YASARA (www.yasara.org, accessed on 20 July 2021) from an amino acid sequence of 859 residues obtained from Uniprot (acc#Q96JP9). The program modeled 1–698 amino acid residues using the human protocadherin 10 ectodomain (PDB: 6gv4) as a structural template. The protein structure containing p.Glu201Gly mutation was equilibrated using molecular dynamics in TIP3P water under periodic boundary conditions with atomic parameters of the YASARA2 forcefield. The simulation was performed for 11 periods (ps) to observe the movement of calcium ions.

## 3. Results

### 3.1. Patient Characteristics

Ten patients from nine unrelated families with retinal dystrophy and *CDHR1* variants were identified. The cohort included six males (60%) and four females (40%). The mean age of the subjects was 45.1 years (range 25.6–62.6 years) at the time of initial examination at the NEI. Participants self-identified their race as White (*n* = 6), Asian (*n* = 2), and African American (*n* = 2).

### 3.2. Genetic Analysis/Molecular Findings

Eleven different *CDHR1* variants were detected and classified based on ACMG criteria in this study cohort (Table 1).

The previously reported variant c.783G > A [10,13,17] was present in four patients. One patient was homozygous for a previously reported frameshift mutation c.1463delG in exon 13 of *CDHR1* [18]. The reported splice site mutation c.1485 + 2T > C was identified in a patient of Puerto Rican descent [19,20]. Three patients of Greek background were found to possess a 7-base pair (bp) deletion in *CDHR1*, c.2522_2528delTCTTCTGA [11], and two of them were found to carry c.783G > A as the second pathogenic variant, whereas the third individual carried the c.115A > G variant. Two African American siblings were homozygous for a novel *CDHR1* variant, c.143C > A.

**Figure 1:**
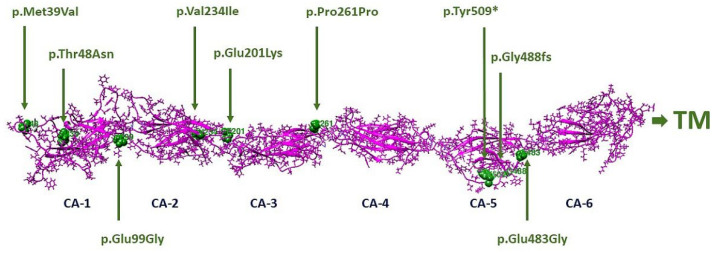
Locations of amino acid changes (green) in the CDHR1 domain structure (pink, residues 1–698). Structural domains were initially localized using SMART: Sequence analysis results for CDHR1_HUMAN (embl.de). Cadherin domains were CA-1 (residues 36–135), CA-2 (136–246), CA-3 (247–353), CA-4 (359–472), CA-5 (473–576), and CA-6 (residues 36–135). The transmembrane helix, TM (702–724), is not shown.

We identified four missense variants (c.115A > G (p.Met39Val); c.143C > A (p.Thr48Asn); c.601G > A (p.Glu201Lys); c.700G > A (p.Val234Ile), one synonymous variant c.783G > A (p.Pro261Pro)), two frameshift variants (c.1463delG (p.G488fs), c.2522_2528delTCTCTGA (p.Ile841Serfs*119)), two variants that affect canonical splice site (c.525 + 1G > A; c.1485 + 2T > C), one nonsense variant (c.1527T > G (p.Tyr509 *)), and one large deletion that included the whole *CDHR1* gene. Mutation locations in the CDHR1 domain structure of the protein from our study cohort are shown in Figure 1.

### 3.3. Clinical Subgroups and Age at First Manifestation

Three patients had the first manifestation of the disease in childhood, while seven were asymptomatic until at least the third decade of life. Four patients had a referral diagnosis of Stargardt macular dystrophy.

Subjects were subdivided based on clinical presentation and full field ERG results: (1) three subjects with presenting symptoms of night vision difficulties who demonstrated undetectable scotopic and photopic ERG responses comprised the RCD group; (2) six subjects with reduced photopic ERG responses comprised the CRD group; and (3) one subject who presented with normal scotopic and subnormal photopic ERG responses demonstrated an isolated bilateral involvement of cone photoreceptors within the macula and was diagnosed with LOMD.

In the RCD group, two unrelated female patients became symptomatic in the third decade of life, while the male subject began to experience first symptoms of vision loss at five years of age. At the time of evaluation, the mean age of this group was 30 ± 3.7 years (median = 30.9 years). These subjects demonstrated rod and cone photoreceptor function loss.

Six patients in the CRD group had a wide age range of disease onset (varying from 10 to 45 years of age). The patients were first evaluated at our clinic in the fifth decade of life. Two male siblings homozygous for the novel *CDHR1* variant, c.143C > A (p.Thr48Asn), showed clinical heterogeneity with the more affected sibling having an onset of symptoms at the age of 10 years, while his brother first noticed vision loss only at the age of 45 years.

The CRD group showed a variation in amplitude reduction and prolongation of implicit times in scotopic and photopic ERG responses (with more severe loss of photopic compared to scotopic ERG).

### 3.4. Clinical Findings

**Figure 2:**
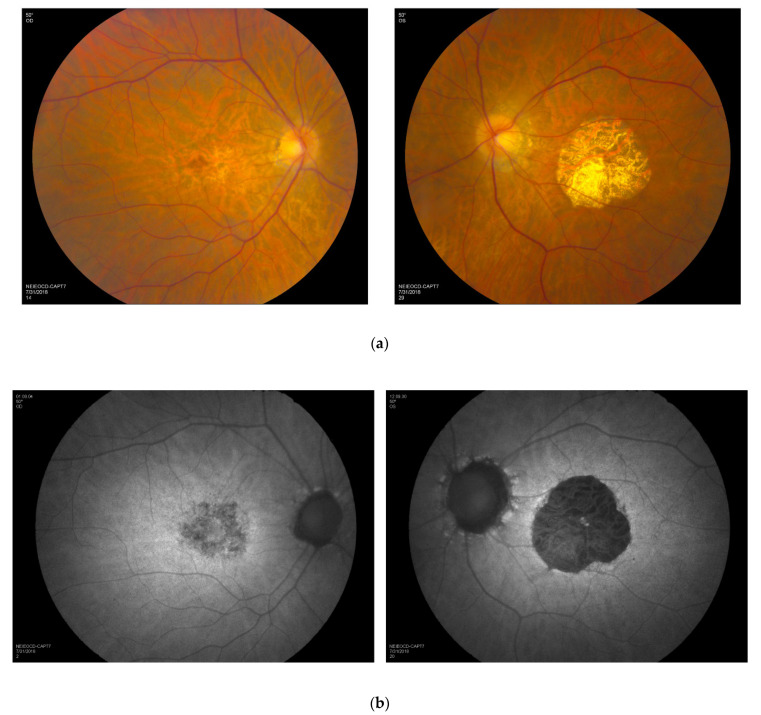
Asymmetry of macular involvement. Images document bilateral areas of macular atrophy with asymmetric disease indicating a greater degree of atrophy in the left eye (with questionably decreased autofluorescence in the right eye and distinctly decreased autofluorescence in the left eye). Color fundus photographs (**a**) and FAF images (**b**) of the right and left eyes of a patient with CRD. FAF, fundus autofluorescence; CRD, cone–rod dystrophy.

Visual acuity loss was the most common presenting symptom. All patients in this cohort had a reduction in visual acuity in at least one eye; two patients in the CRD group showed normal values of visual acuity in one eye. Visual acuity ranged from 20/16 to 20/2000 (mean LogMAR 0.8 or 20/125). The RCD group demonstrated the most preserved visual acuity, ranging from 20/25 to 20/63. Interocular difference in visual acuity related to retinal dystrophy was observed in three patients, and a prominent asymmetry of macular atrophy was present in two individuals (Figure 2).

Measurement of visual fields revealed a considerable variation in defects in the cohort. Three patients diagnosed with RCD reported peripheral vision loss. One of them demonstrated tunnel vision with severe constriction of visual fields with V4e isopter (continuous horizontal diameter less than 20 degrees). The third subject with RCD demonstrated better-preserved visual fields with small absolute bilateral paracentral scotomas and the inability to see the I1e target. The subjects who were diagnosed with CRD or LOMD had evidence of central scotomas that mirrored the degree of macular atrophy. Color vision was significantly impaired in all patients. One subject in the CRD group and one subject in the RCD group were unable to distinguish colors. Color vision defects were more severe in patients with larger areas of macular atrophy. Clinical findings are summarized in Table 2.

### 3.5. Retinal Imaging

**Figure 3:**
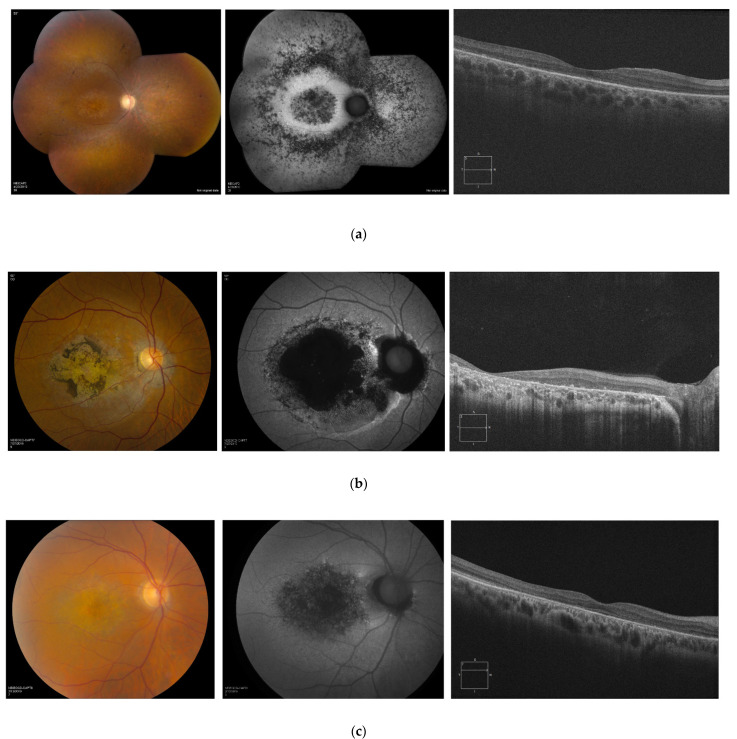
Color fundus photographs, FAF images, and macular OCT scans of patients with (**a**) RCD, (**b**) CRD, and (**c**) LOMD. Note: (**a**) the early macular involvement in a 28-year-old female with RCD diagnosis; (**b**) the clinical findings of an extensive macular atrophy and pigmentation with a reduction in photopic and mildly subnormal scotopic ERG responses in a patient with CRD; (**c**) bilateral macular atrophy and diffuse generalized hypoautofluorescence, normal rod with reduced cone ERG responses in a patient with LOMD. FAF, fundus autofluorescence; OCT, optical coherence tomography; RCD, rod–cone dystrophy; CRD, cone–rod dystrophy; LOMD, late-onset macular dystrophy; ERG, electroretinography.

An examination of the retina in addition to a review of color fundus photography, FAF and OCT demonstrated evidence of posterior pole atrophy in all members of the cohort and peripheral abnormalities in RCD patients (Figure 3).

**Figure 4:**
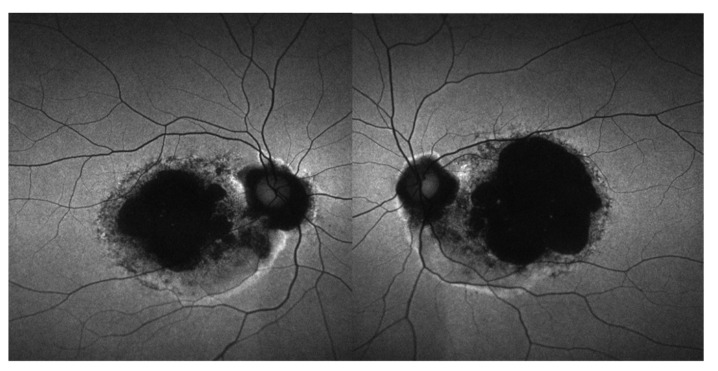
FAF images of the right and left eyes of the patient with early onset CRD harboring a novel homozygous c.143C > A (p.Thr48Asn) *CDHR1* variant. The panel shows coalescence of hypoautofluorescence with surrounding areas of irregular hyperautofluorescence in the macular area bilaterally. FAF, fundus autofluorescence; CRD, cone–rod dystrophy.

The fundi of patients diagnosed with RCD were characterized by bilateral vascular attenuation, scattered peripheral pigmentary changes, and diffuse RPE atrophy. In this group of patients, OCT B-scans through the macula demonstrated diffuse loss of photoreceptors and thinning of the retina (Figure 3a). Retina appearance in patients with CRD and LOMD was marked by mild retinal vasculature attenuation, extensive macular atrophy, and pigment deposition within the macular region (Figure 3b,c). In three individuals with CRD, the FAF images showed coalescence of hypoautofluorescence in the macular area bilaterally, with surrounding areas of irregular hyperautofluorescence delineating the edge of the lesions (Figure 4).

In patients with CRD, macular OCT scans demonstrated a generalized retinal thinning with diffuse loss of photoreceptor ellipsoid zone, especially prominent within the fovea. Involvement of the macular region was noted in the entire cohort (often in a bull’s eye maculopathy appearance).

### 3.6. Longitudinal Data

Longitudinal data were available on 7 out of 10 subjects in the cohort. Most individuals had yearly follow-up visits with repeat visual function evaluation. One male subject from the RCD group had a 4-year period of follow-up visits. He demonstrated a decline in visual acuity from 20/32 to 20/50 in the right eye and from 20/25 to 20/32 in the left eye. In the CRD group, five subjects were evaluated annually. One male subject was followed over an 8-year period. He demonstrated a severe decline in visual function from 20/63 to “counting fingers” in the right eye and stable visual acuity in the left eye (hand motion from the time of the first presentation). Four participants from the CRD group had relatively stable visual function within 3-, 5-, and 8-year follow-up periods. The patient with LOMD was followed for 13 years. Within this timeframe, her visual acuity changed from 20/100 in the right eye and 20/80 in the left eye to 20/200 in both eyes.

### 3.7. Molecular Modeling

**Figure 5:**
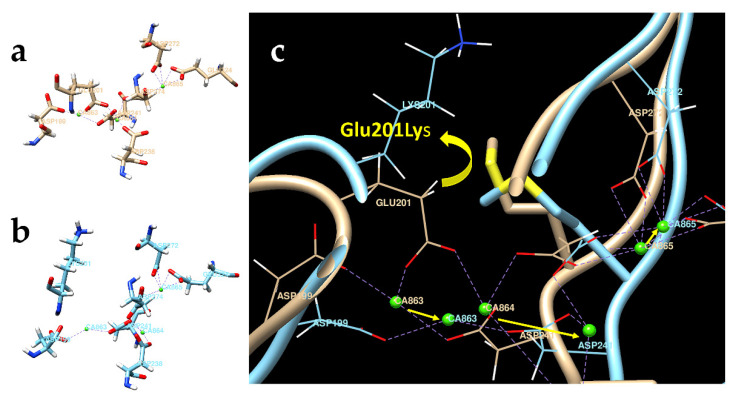
The pathogenic variant Glu201Lys is predicted to decrease Ca^2+^ binding to the CDHR1 protein. Glu201Lys is confined in the interface area between cadherin domains CA-2 and CA-3. Ca^2+^ is shown by green spheres. (**a**) In the normal CDHR1 protein structure (beige), Ca^2+^-binding sites are formed by negatively charged residues. (**b**) In the altered CDHR1 protein p.Glu201Lys (blue), the replacement of negatively charged Glu with positively charged Lys decreases Ca^2+^ binding. (**c**) The superposition of the normal CDHR1 and the p.Glu201Lys proteins was refined using 11 ps computer simulation. Yellow arrows show the movement of Ca^2+^ ions.

In the current study, we proceeded with a computer simulation model to study the effect of the *CDHR1* missense variants on protein structure and function. We have observed an interesting finding indicating that c.601G > A (p.Glu201Lys) variant affects interference of the Ca^2+^-binding domain of the CDHR1 protein (Figure 5).

In the protein Glu201Lys, the loss of electrostatic interaction between Glu201(−) and Ca^2+^ ions and the repulsive interaction between positively charged Lys (+) and Ca^2+^ lead to decreased calcium ion binding. This is confirmed by 11 ps computer simulation, which demonstrated the movement of calcium ions out of calcium-binding sites (calcium ion CA863 has moved at 2 Å, CA864 at 8 Å, and CA865 at 1 Å). We expect a similar effect for proteins p.Glu99Gly and p.Glu483Gly localized in the CA-1/CA-2 and CA-5/CA-6 interdomain interfaces, respectively (Supplementary Table S1). The loss of calcium ions could lead to the loss of chain rigidity and increased flexibility in altered CDHR1 proteins.

## 4. Discussion

In humans, variants in *CDHR1* are associated with autosomal recessive retinal dystrophies such as CRD, RCD, and LOMD [10,11,12,13,21]. In our cohort, we identified six individuals with CRD, three individuals with RCD, and one individual with LOMD based on clinical presentation and ERG findings. In all three groups of patients, macular atrophy was a characteristic feature.

The first report of *CDHR1*-associated retinal dystrophy, i.e., CRD, identified in a small consanguineous family from the Faroe Islands was published by Ostergaard E. et al. in 2009 [9]. Over the past decade, additional studies have reported novel variants in *CDHR1* and have demonstrated the correlation between phenotype and genotype in patients with *CDHR1* retinal dystrophies [22]. We expand the phenotypic spectrum associated with molecular changes in the *CDHR1* gene including RCD, CRD, and LOMD. In our cohort, six individuals with CRD were found to harbor missense variants c.143C > A (p.Thr48Asn), c.601G > A (p.Glu201Lys), and c.700G > A (p.Val234Ile); one synonymous variant c.783G > A (p.Pro261Pro); one nonsense variant c.1527T > G (p.Tyr509*); two variants that affect canonical splice sites, c.525 + 1G > A and c.1485 + 2T > C; and one frameshift deletion variant c.2522_2528delTCTCTGA (p.Ile841Serfs*119) in *CDHR1*. Three individuals with RCD were identified to carry the synonymous variant c.783G > A (p.Pro261Pro), frameshift deletion variants c.1463delG (p.G488fs) and c.2522_2528delTCTCTGA (p.Ile841Serfs*119) in *CDHR1*, and one large deletion that included the elimination of the whole *CDHR1*. The individual with LOMD harbored one synonymous variant c.783G > A (p.Pro261Pro) and the frameshift deletion variant c.2522_2528delTCTCTGA (p.Ile841Serfs*119) in *CDHR1*.

A clear correlation between genotype and phenotype is difficult to establish due to the small cohort size and multiplicity of *CDHR1* pathogenic variants carried by subjects in all three groups in our study. However, all individuals clinically present with early macular involvement, even in the setting of RCD and relatively preserved visual acuity. Of note, the degree of macular atrophy does not seem to correlate with age in this cohort; however, age of onset might be a prognostic factor for disease progression and severity.

Similar clinical findings were observed in patients with autosomal recessive *PROM1* retinal dystrophy [23] and in patients who harbored a *cis*-acting recessive allele in *CDHR1* along with the *RGR* mutation [24]. Stone et al. estimated that the total number of individuals in the United States who are affected by retinal dystrophies associated with mutations in *CDHR1* could be over 700, with an incidence rate of nine cases per year [25]. These observations may help guide prognosis, family-decision making, and access to clinical trials and treatments.

Variants in *CDHR1* have been described in individuals with autosomal recessive retinal degeneration from diverse ancestry and ethnic backgrounds [19,20,26,27,28]. Here, we report a novel homozygous *CDHR1* variant, c.143C > A (p.Thr48Asn), in two related patients of African American descent with CRD, and a 7-bp deletion in *CDHR1*, c.2522_2528delTCTTCTGA (p.Ile841Serfs*119), in three patients of Greek background. We hypothesize that this 7-bp deletion may point to a possible founder effect. We identify the need for larger studies, likely multicenter, that hopefully will draw from diverse populations to better understand molecular aspects that correlate with the phenotype in *CDHR1* retinopathy.

Tiwari et al. have reported the prediction of the structure of CDHR1 protein aligned to a *CDHR1* pathogenic variant, c.398C > G (p.Pro133Arg), in a patient with retinitis pigmentosa by molecular modeling [29]. In our study, we applied a molecular modeling approach and observed an interesting finding in *CDHR1* variant c.601G > A (p.Glu201Lys). We hypothesize that in the altered CDHR1 protein, the loss of electrostatic interaction between negatively charged amino acid Glu201(−) and Ca^2+^ ions, and the repulsive interaction between positively charged Lys (+) and Ca^2+^ may lead to interference of the Ca^2+^-binding domain within the protein. The loss of Ca^2+^ ions can lead to the instability of a protein, thus affecting the junction between the inner and outer segments of rod and cone photoreceptor cells.

The spectrum of *CDHR1* retinopathy includes photoreceptor degenerations, CRD, and RCD. LOMD could be characterized as a milder spectrum-extending finding within the *CDHR1* retinopathy presentation. In our cohort, similar genotype combinations are seen in the clinical subgroups preventing a robust genotype–phenotype correlation. Macular atrophy is noted early in the disease presentation and can be asymmetric with implications for visual function and future clinical trial planning. This cohort is drawn from the diverse racial and ethnic backgrounds, highlighting the need for larger studies with a better appreciation of disease findings and correlations.

## 5. Conclusions

The present study highlights the phenotypic spectrum of autosomal recessive retinal dystrophies associated with molecular changes in the *CDHR1* gene including RCD, CRD, and LOMD. The results confirm that individuals with molecular changes in *CDHR1* present with early macular involvement, even in the setting of RCD. The degree of macular atrophy does not seem to correlate with age in this small cohort; however, age of onset might be a prognostic factor for disease progression and severity. Asymmetry in the degree of macular involvement is noted in two patients. Further studies are required to better understand correlation between genotype and phenotype in individuals with *CDHR1*-associated retinal dystrophies.

## Figures and Tables

**Table 1:** Summary of all *CDHR1* variants identified in this study according to ACMG standards [14].

*CDHR1* Variant	Protein Change	ACMG Classification
c.115A > G	p.Met39Val	VUS (PM2)
c.143C > A	p.Thr48Asn	VUS (PM2)
c.525 + 1G > A	Abnormal splicing	Pathogenic (PVS1, PM2, PP5)
c.601G > A	p.Glu201Lys	VUS (PS4-moderate, PM2, PP3)
c.700G > A	p.Val234Ile	VUS (PM2, BP4)
c.783G > A	p.Pro261=	Pathogenic (PS4, PS3, PM3, PP3)
c.1463delG	p.Gly488fs	Pathogenic (PVS1, PS4-moderate, PM2)
c.1485 + 2T > C	Abnormal splicing	Pathogenic (PVS1, PS4-moderate, PS3, PM3)
c.1527T > G	p.Tyr509*	Pathogenic (PVS1, PS4-moderate, PM2)
c.2522_2528delTCTCTGA	p.Ile841Serfs*119	Pathogenic (PVS1, PS4-moderate, PM2)
7.38 Mb del including *CDHR1*	-	Pathogenic (PVS1, PM2, PM3)

**Table 2:** Summary of clinical findings in our *CDHR1* cohort. Each row in the table represents a single patient. Scotopic 0dB b-wave and photopic 30 Hz flicker ERG amplitudes are presented as an average value of ERG response amplitudes from the right and left eyes of each patient (lower limit of normal is 373 µV for the scotopic 0 dB b-wave amplitude and 66 µV for the photopic 30 Hz flicker ERG amplitude). RCD, rod–cone dystrophy; CRD, cone–rod dystrophy; LOMD, late-onset macular dystrophy; F, female; M, male; BCVA, best corrected visual acuity; OD, right eye; OS, left eye; CF, counting fingers; GVF, Goldmann visual field perimetry; HVF, Humphrey visual field perimetry; ERG, electroretinography.

Phenotype	Ethnicity/Race	Gender	Age at First Symptoms (years)	Age at First NEI Visit (years)	BCVA OD	BCVA OS	GVF OD	GVF OS	D-15 OD (Number of Errors)	D-15 OS (Number of Errors)	Scotopic 0 dB b-Wave (µV)	Photopic 30 Hz Flicker (µV)	*CDHR1* Variant 1	*CDHR1* Variant 2
RCD	Caucasian	F	28	33	20/50	20/63	severely constricted	severely constricted	multiple	multiple	0	0	c.783G > A	7.38 Mb del including *CDHR1*
RCD	Greek	F	~26	31	20/50	20/50	constricted	constricted	unable to distinguish colors	unable to distinguish colors	0	12	c.783G > A	c.2522_2528delTCTCTGA
RCD	Bangladeshi	M	~5	26	20/32	20/25	central scotoma	central scotoma	multiple	multiple	0	5	c.1463delG	c.1463delG
CRD	Chinese	M	35	49	CF < 8″	CF < 8″	constricted	constricted	multiple	multiple	162	31	c.601G > A	c.700G > A
CRD	Romanian	M	~15	49	20/20	20/500	central scotoma	central scotoma	two	multiple	256	37	c.783G > A	c.1527T > G
CRD	Puerto Rican	F	~30	49	20/63	20/63	central scotoma	central scotoma	unable to distinguish colors	unable to distinguish colors	278	11.5	c.1485+2T > C	c.525+1G > A
CRD	African American	M	~10	45	20/200	20/200	central scotoma	central scotoma	three	no errors	236	44.5	c.143C > A	c.143C > A
CRD	African American	M	45	54	20/400+2	20/400+3	central scotoma (HVF)	central scotoma (HVF)	two	multiple	247	22	c.143C > A	c.143C > A
CRD	Greek	M	~45	63	20/16	20/63	central scotoma	central scotoma	multiple	multiple	251	70.5	c.115A > G	c.2522_2528delTCTCTGA
LOMD	Greek	F	~40	53	20/100	20/80	central scotoma	central scotoma	multiple	multiple	363	70	c.783G > A	c.2522_2528delTCTCTGA

## Data Availability

All relevant data are within the paper and its Supporting Files.

## Supplementary Materials

The following supporting information can be downloaded at: https://www.mdpi.com/article/10.3390/genes13050925/s1, Table S1: The functional effect of genetic missense mutations in this study.
