# Supplementary material for: Clinical Phenotypes of CDHR1-Associated Retinal Dystrophies
Source: Genes (Basel). 2022 May 22;13(5):925. doi: 10.3390/genes13050925 (PMC9140808; doi:10.3390/genes13050925)
Supplement: Supplementary file 1 [file genes-13-00925-s001.zip › genes-1697188-supplementary.pdf]

Supplementary Table S1. The functional effect of genetic missense mutations in this study.

***CDHR1* Variant**	**Mutation**	**Motif Location**	**Effect of Mutation**
c.115A>G	M39V	N-terminal	N/A
c.143C>A	T48N	N-terminal	N/A
c.296A>G	E99G	CA1	Ca+ binding
c.601G>A	E201K	CA2	Ca+ binding
c.700G>A	V234I	CA2	N/A
c.1373T>A	V458D	CA4	N/A
c.1448A>G	E483G	CA4-CA5	Ca+ binding
c.1700T>C	L567P	CA5	Domain-folding
c.1720C>G	P574A	CA5	Domain-folding
c.2027T>A	L676N	CA6	N/A
c.2108G>A	G703D	TM	N/A
c.2203C>A	P735T	TM	N/A
